# SGLT2 inhibitors in kidney transplant recipients: metabolic changes, potassium-related safety and implications for albuminuria assessment

**DOI:** 10.3389/frtra.2026.1927374

**Published:** 2026-08-18

**Authors:** Lino Henkel, Lisa Ingmanns, Florian Westphal, Amélie F. Menke, Hermann Pavenstädt, Ulrich Jehn, Göran R. Boeckel, Stefan Reuter

**Affiliations:** 1 Department of Medicine D, University Hospital Münster, Münster, Germany; 2 Department of Nephrology, University Hospital Zurich, Zurich, Switzerland; 3 Nephrologisches Zentrum Emsland, Lingen, Germany

**Keywords:** albuminuria, graft function, hyperkalemia, kidney transplantation, metabolic effects, renal transplantation, SGLT2 inhibitor, urinary creatinine

## Abstract

**Background:**

Sodium-glucose cotransporter-2 inhibitors (SGLT2i) are established therapies in chronic kidney disease (CKD), but comparative data in kidney transplant recipients (KTR) remain limited.

**Methods:**

We retrospectively matched 100 KTR receiving SGLT2i to 100 controls not receiving SGLT2i and assessed body mass index (BMI), HbA1c, urine albumin-to-creatinine ratio (uACR), estimated glomerular filtration rate (eGFR), serum potassium, RAAS inhibitor use, blood pressure, and safety outcomes at 6 and 12 months. Furthermore, we compared the Jaffé and enzymatic methods to examine the effect of SGLT2i-associated glucosuria on urinary creatinine measurements and the resulting uACR.

**Results:**

BMI decreased in the intervention group and increased in controls; the overall adjusted between-group difference across follow-up remained statistically significant. Descriptive between-group differences in HbA1c were observed at 6 and 12 months, but were not confirmed after multivariable adjustment. Neither uACR nor eGFR differed significantly at 12 months, despite an initial decline in eGFR at 6 months. Serum potassium remained stable over time. Hyperkalemia was associated with lower eGFR, but not with SGLT2i treatment, and RAAS inhibitor persistence did not differ between groups. No statistically detectable differences or clear new safety signals were observed for blood pressure, urinary tract infections, cardiovascular events, or hospitalizations during follow-up. Jaffé-based creatinine measurements were lower than enzymatic measurements, increasing calculated uACR by approximately 8.5% (*p* < 0.001), with rare albuminuria stage reclassification.

**Conclusions:**

SGLT2i use was associated with favorable metabolic changes, particularly a reduction in BMI, without evidence of increased potassium-related risk. No improvement in graft function or albuminuria was observed over 1 year. SGLT2i-associated glucosuria systematically increased Jaffé-based uACR estimates, although clinically relevant albuminuria reclassification was uncommon.

## 1 Introduction

Originally developed as antihyperglycemic agents, sodium–glucose cotransporter-2 inhibitors (SGLT2i) represent a prominent example of drug repurposing toward chronic kidney disease (CKD) therapy because their disease-modifying effect in terms of kidney and cardiovascular benefits extends beyond glycemic control (1). They slow the decline of kidney function, reduce albuminuria, and improve cardiovascular outcomes in a wide range of CKD patients (2–5). However, kidney transplant recipients (KTR) were excluded from these landmark studies, and data in this population remain limited. Reflecting this evidence gap, the 2022 KDIGO guideline for diabetes management in CKD stated that the recommendation to use SGLT2i does not apply to KTR, mainly because of limited data and unresolved concerns regarding safety in the setting of immunosuppression (6).

Since then, several retrospective cohorts, one randomized placebo-controlled trial, and recent systematic reviews have suggested that SGLT2i can be used in selected KTR with an overall acceptable short-term safety profile. At the same time, follow-up remains short in most studies, event numbers are limited, and firm conclusions regarding graft survival, cardiovascular events, or long-term renal benefit remain difficult to draw (7–13). In addition, Chapman et al. highlighted a potential bias introduced by SGLT2i-associated glucosuria on urine creatinine measurement and its impact on albumin-to-creatinine ratio (uACR), potentially leading to misclassification of albuminuria stage (14).

Among KTR, the most consistent findings thus far are related to metabolic endpoints, particularly improved glycemic control and modest weight loss. However, effects on graft function and albuminuria have been less consistently demonstrated across studies (9–12, 15). Beyond glycemic and weight-related effects, SGLT2 inhibition may also influence potassium, magnesium, phosphate, and other components of electrolyte and mineral homeostasis through natriuresis, osmotic diuresis, and altered distal tubular solute delivery (16–19). Recent routine-care data in non-transplant patients with diabetes mellitus (DM) further suggest that SGLT2i may reduce the risk of hyperkalemia, although this has not consistently translated into improved maintenance of renin-angiotensin-aldosterone system inhibitor (RAAS inhibitor) therapy. Whether similar potassium-related safety patterns occur after kidney transplantation remains unclear, because potassium balance in KTR is additionally influenced by graft function, calcineurin inhibitor exposure, RAAS blockade, and transplant-specific comorbidities (20).

Accordingly, our aim was to evaluate the effects of SGLT2i therapy on graft function, metabolic parameters, potassium-related safety, RAAS inhibitor use, and safety outcomes in KTR using a matched control group not receiving SGLT2i therapy. Additionally, we examined the potential impact of SGLT2i-associated glucosuria on urine albumin-to-creatinine ratio (uACR) measurement by comparing Jaffé-based and enzymatic urinary creatinine assays. By combining a matched clinical comparison with exploratory analyses of potassium behavior, RAAS inhibitor use, and uACR assay sensitivity, we aimed to provide clinically relevant real-world evidence on SGLT2i use after kidney transplantation (9, 11, 12).

## 2 Materials and methods

### 2.1 Study design

We retrospectively analyzed 100 adult kidney transplant patients treated with an SGLT2i (intervention group, IG) including patients with (*n* = 63) and without (*n* = 37) DM, and matched them to 100 kidney transplant patients not treated with an SGLT2i (control group, CG). All patients underwent transplantation and follow-up at our center. SGLT2i exposure was ascertained by manual review of medication records in the electronic hospital information system. For the intervention group, SGLT2i use was documented in the medication record, with the last available visit before treatment initiation defined as baseline. SGLT2i use during the subsequent 12-month follow-up was verified by manual review of medication records. Control patients were required to have no documented SGLT2i use at the assigned baseline time point or during the corresponding 12-month observation period. No patient crossed over between the IG and CG during follow-up.

Control patients were matched 1:1 to SGLT2 inhibitor-treated kidney transplant recipients using minimum-cost bipartite matching based on the Hungarian algorithm. Pairwise Euclidean distances were calculated using age at transplantation, time since transplantation, and binary-coded sex. In SGLT2 inhibitor-treated recipients, time since transplantation was calculated from the transplantation date to the pretreatment baseline date. In potential controls, time since transplantation was assigned according to the latest available predefined post-transplant follow-up time point. Because treatment initiation and corresponding baseline assessments occurred at different time points in routine clinical care, the observation periods of each patient and the respective matched control were not necessarily identical in calendar time.

The observation period covered the first year after SGLT2i initiation, with assessments at baseline, 6 months and 1 year as part of routine follow-up. Analyses used available-case data; no participant was excluded solely because a follow-up measurement was missing. Follow-up measurements obtained after permanent discontinuation of SGLT2i therapy were not included in the longitudinal analyses; patients were therefore censored at the time of treatment discontinuation. Patient records were anonymized prior to analysis.

The study was approved by the local ethics committee (Ethik Kommission der Ärztekammer Westfalen-Lippe und der Medizinischen Fakultät der Universität Münster, No. 2014-390-f-N).

### 2.2 Outcomes

The study was conceived to address graft function and albuminuria; changes in estimated glomerular filtration rate (eGFR, CKD-EPI formula, mL/min/1.73 m^2^) and log10-transformed urine albumin-to-creatinine ratio (uACR, mg/g creatinine) were therefore considered the principal clinical outcomes. Secondary clinical outcomes included body mass index (BMI, kg/m^2^), HbA1c (%), serum potassium (mmol/L), blood pressure (mean arterial pressure, MAP, mmHg), hyperkalemia, RAAS inhibitor persistence or initiation, and safety parameters. The comparison of Jaffé-based and enzymatic urinary creatinine measurements under SGLT2i-associated glucosuria was considered an exploratory laboratory-methodological analysis. No formal multiplicity correction was applied; effect estimates and 95% confidence intervals were emphasized, and *p*-values for secondary and exploratory outcomes were considered hypothesis-generating. Safety endpoints comprised cardiovascular events, hospitalization, marked decline in graft function (defined as eGFR loss > 30%), urinary tract infection (UTI), and genital infection. Patients with chronic antibody-mediated rejection were excluded from the principal uACR analysis because this condition may substantially confound albuminuria assessment; this affected 10 matched pairs. A sensitivity analysis including these pairs was also performed.

### 2.3 Comparison of urinary creatinine

During routine follow-up visits at our center, 128 consecutive urine samples were collected from 100 KTR receiving SGLT2i therapy (including 28 repeat samples), all with glucosuria ≥ 55 mmol/L (≙ 991 mg/dL). Urine creatinine was measured in parallel using an enzymatic assay and the Jaffé method (Roche Cobas C Analyzer, Roche, Basel, Switzerland). The Jaffé method is based on a colorimetric reaction between creatinine and alkaline picrate and is susceptible to interference by non-creatinine chromogens, including glucose. In contrast, enzymatic assays are considered more specific and less affected by glucosuria (21). These measurements were used to assess the potential impact of SGLT2i-associated glucosuria on creatinine-based uACR calculation.

### 2.4 Statistical analysis

Continuous variables are reported as mean ± SD or median (IQR), and categorical variables as number (percentage). Because uACR values were right-skewed, uACR was log10-transformed before inferential analyses. Descriptive uACR values are presented on the original scale to facilitate clinical interpretation, whereas statistical comparisons of uACR were performed using log10-transformed values. Normally distributed continuous variables were compared using independent-samples t-tests, whereas non-normally distributed continuous variables were compared using Mann–Whitney U tests. Categorical variables were compared using *χ*^2^ tests or Fisher's exact tests, as appropriate. For the unadjusted longitudinal analyses of clinical parameters, individual changes from baseline to 6 and 12 months were calculated among participants with paired measurements and compared between groups using two-sided Mann–Whitney U tests. Descriptive values presented in the main text and figures were based on all available observations at each time point, with the corresponding number of observations reported for each assessment. Complete-case descriptive analyses restricted to participants with measurements available at baseline, 6 months, and 12 months are provided in the Supplementary Material. To address residual confounding due to baseline imbalances, adjusted sensitivity analyses were performed for BMI, HbA1c, eGFR, log10-transformed uACR, and MAP. Overall between-group differences during follow-up were estimated using linear mixed-effects models including treatment group, follow-up time, the group-by-time interaction, and outcome-specific baseline covariates. Time-specific ANCOVA models were additionally performed at 6 and 12 months using the same covariate sets. Full model specifications, sample sizes, adjusted estimates, and 95% confidence intervals are provided in Supplementary Tables S6 and S7. Given the limited number of repeated measurements for some outcomes, particularly HbA1c, the multivariable models were considered sensitivity analyses rather than confirmatory analyses.

In addition, exploratory analyses of serum potassium, hyperkalemia, and RAAS inhibitor use were performed. Hyperkalemia was defined as serum potassium >5.0 mmol/L. Repeated serum potassium measurements were analyzed using a linear mixed model with fixed effects for group, time, and the group-by-time interaction. Repeated hyperkalemia measurements were analyzed using generalized estimating equations to account for within-patient correlation. Patient-level occurrence of hyperkalemia during follow-up and RAAS inhibitor persistence or initiation were assessed using logistic regression models.

Because of the retrospective design and incomplete availability of repeated measurements in routine follow-up, no imputation of missing values was performed. All analyses were therefore based on available data. A two-sided *p*-value < 0.05 was considered statistically significant. Analyses were performed in SPSS 31 (IBM) and charts were created using GraphPad Prism version 8 (GraphPad Software).

## 3 Results

### 3.1 Study population

Table 1 summarizes the baseline characteristics. Age, sex, transplant duration, donor type distribution, blood pressure, serum potassium, serum creatinine, eGFR, CKD stage, and albuminuria stage were broadly comparable between groups. However, BMI, HbA1c, RAAS inhibitor use and the prevalence of DM were higher in the IG, whereas pre-transplant listing time was longer in the CG (BMI: *p* = 0.016; HbA1c: *p* < 0.001; RAAS inhibitor use: *p* = 0.015; DM: *p* < 0.001; pre-transplant listing time: *p* = 0.004).

**Table 1:** Baseline characteristics of the study population.

Variable	Unit/summary	Intervention Group (IG) *n* = 100	Control Group (CG) *n* = 100	Statistical test	p
Age at transplantation	years, mean ± SD	49.6 ± 14.56	50.1 ± 14.16	t-test	0.806
Female sex	n	25	25	*χ*^2^ test	1.000
Pre-transplant waiting-list time	months, median, IQR	64.16, 24.44–100.08	88.38, 47.62–149.97	Mann–Whitney U	0.004
Deceased donor	n/N (%)	64/95 (67.4)	69/98 (70.4)	χ^2^ test	0.648
Time since transplantation at baseline	months, median, IQR	46.77, 11.15–117.51	32.72, 11.92–149.86	Mann–Whitney U	0.063
BMI	kg/m^2^, mean ± SD	27.60 ± 4.52	26.1 ± 4.18	t-test	0.016
Predominant cause leading to ESKD hypertension diabetes polycystic kidney disease reflux nephropathy glomerulonephritis or Alport syndrome focal segmental sclerosis interstitial nephritis vasculitis other or unknown	n	9 7 12 4 35 6 1 1 25	9 6 12 2 30 4 2 0 35	Descrip-tive only	
Diabetes mellitus NODAT Type 2	n/N (%)	63/99 (63.6) 43/99 (43.4) 20/99 (20.2)	30/97 (30.9) 21/97 (21.6) 9/97 (9.3)	χ^2^ test	<0.001 <0.001 0.031
Glucose-lowering therapy None insulin only non-insulin antidiabetics only both	n	51 22 20 7	84 12 3 1	Fisher's exact test	<0.001
HbA1c	%, mean ± SD	6.89 ± 1.51	5.81 ± 0.82	t-test	<0.001
Cardiovascular diseases Hypertension Stroke Coronary disease Peripheral artery disease Atrial fibrillation	n	95 4 29 2 7	90 0 20 7 10	Descrip-tive only	
Blood pressure at last outpatient clinic systolic diastolic Hypertension grades normal high-normal grade 1 grade 2 grade 3 no data	mmHg, mean ± SD n	141.73 ± 16.44 86.04 ± 14.02 20 14 35 18 5 8	138.57 ± 16.85 83.73 ± 12.05 24 18 41 5 4 8	t-test t-test Fisher's exact test	0.199 0.230 0.054
RAAS inhibitor use ACE inhibitor/AT1 blocker plus aldosterone antagonist none	n	72 2 26	52 6 42	Fisher's exact test	0.015
Serum potassium Patients with hyperkalemia > 5 mmol/L	mmol/L, mean ± SD n	4.32 ± 0.60 11	4.40 ± 0.52 12	t-test χ^2^ test	0.307 0.825
uACR A1 (<30) A2 (30–300) A3 (>300) Missing	mg/g creatinine, median, IQR n	49.32, 23.50–163.97 33 51 12 4	35.54, 19.65–86.12 44 42 12 2	Mann–Whitney U χ^2^ test	0.073 0.298
Serum creatinine	mg/dL, mean ± SD	1.59 ± 0.50	1.71 ± 0.60	t-test	0.218
eGFR G1 G2 G3a G3b G4 G5	CKD-EPI, mL/min/1.73 m^2^, mean ± SD n	49.78 ± 16.64 4 21 34 31 10 0	47.43 ± 17.86 4 20 27 34 15 0	t-test Fisher's exact test	0.336 0.748
LDL	mg/dL, mean ± SD	95.72 ± 39.48	98.19 ± 33.12	t-test	0.710

Baseline demographic and clinical characteristics are presented for the Intervention Group (IG) and Control Group (CG). Continuous variables are presented as mean ± standard deviation (SD) or median (interquartile range, IQR), as appropriate, and categorical variables as numbers. As each study group comprised 100 participants, counts correspond to percentages unless data were missing. *P*-values reflect between-group comparisons using the statistical test indicated. Variables comprising multiple descriptive subcategories were not formally compared between groups unless otherwise indicated. Denominators may vary between variables because of missing data.

BMI, body mass index; CKD-EPI, Chronic Kidney Disease Epidemiology Collaboration; eGFR, estimated glomerular filtration rate; ESKD, end-stage kidney disease; IQR, interquartile range; LDL, low density lipoprotein; NODAT, new-onset diabetes after transplantation; SD = standard deviation; uACR, urine albumin-to-creatinine ratio.

Regarding graft-related parameters, the baseline eGFR was similar between groups (49.78 ± 16.64 vs. 47.43 ± 17.86 mL/min/1.73 m^2^, *p* = 0.336), as was the distribution of CKD stages (*p* = 0.748). There was a non-significant trend toward higher baseline uACR values in the IG (*p* = 0.073), though there was no significant difference in albuminuria stage distribution between groups (*p* = 0.298).

Metabolic parameters differed at baseline: BMI was 27.60 ± 4.52 kg/m^2^ in the IG vs. 26.10 ± 4.18 kg/m^2^ in the CG, and HbA1c was 6.89 ± 1.51% vs. 5.81 ± 0.82%, respectively. These differences were accompanied by a higher prevalence of DM in the IG. Otherwise, cardiovascular comorbidities and hypertension grades were similarly distributed between groups.

### 3.2 Study outcomes

#### 3.2.1 Principal graft-related outcomes

**Figure 1:**
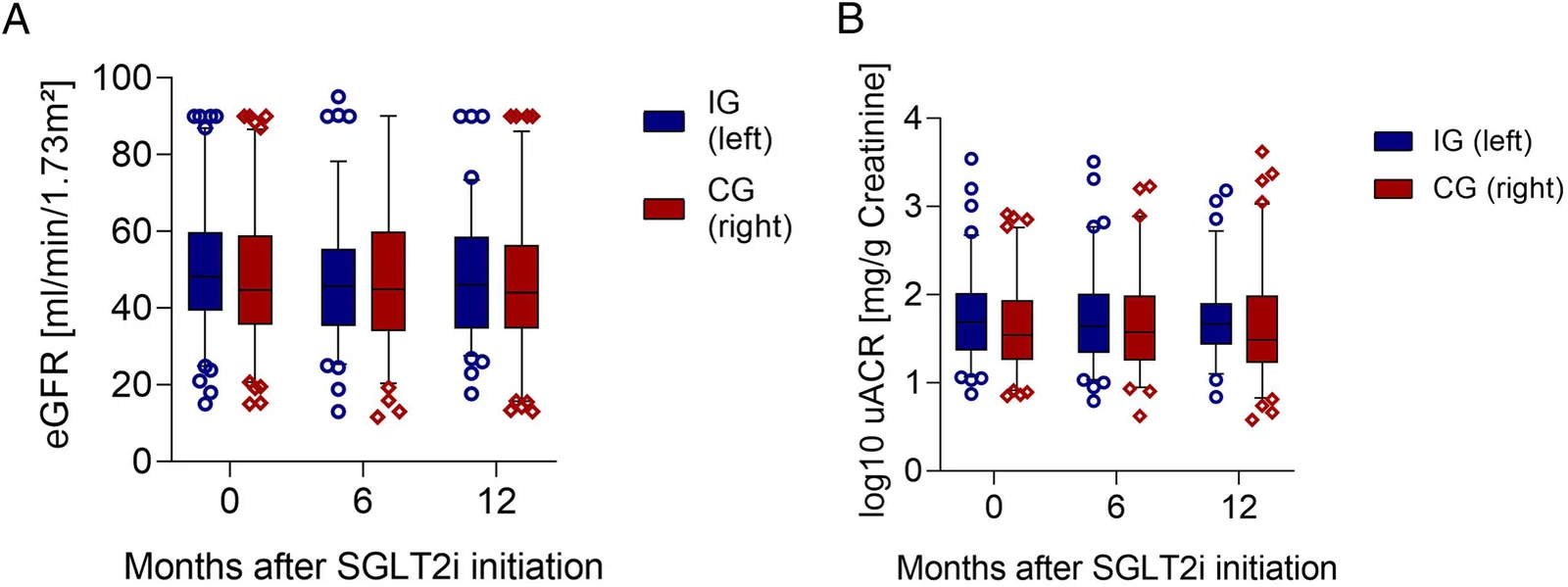
eGFR and uACR over time. eGFR **(A)** and uACR **(B)** over time in the IG (blue, left) and CG (red, right). Boxes indicate the interquartile range (25th–75th percentile), horizontal lines indicate the median, whiskers indicate the 5th–95th percentile, and individual points outside the whiskers represent outliers. In the unadjusted matched-cohort analysis, the between-group difference in eGFR change from baseline was statistically significant at six months, but not at 12 months. Adjusted sensitivity analyses did not demonstrate a significant overall or time-specific baseline-adjusted between-group difference in eGFR during follow-up. No significant between-group differences in uACR change from baseline were observed in either the unadjusted or adjusted analyses. The *y*-axis in **(B)** is displayed on a logarithmic scale because of the skewed distribution of uACR values; inferential analyses of uACR were performed using log10-transformed values.

Figure 1A shows changes in eGFR over time. After six months, the mean eGFR in the IG declined from 49.8 ± 16.6 to 47.7 ± 16.2 mL/min/1.73 m^2^, whereas it remained largely unchanged in the CG. The between-group difference in change from baseline to 6 months was statistically significant (*p* = 0.004). However, at 12 months, mean eGFR was 47.8 ± 15.5 mL/min/1.73 m^2^ in the IG and 46.3 ± 18.3 mL/min/1.73 m^2^ in the CG; the between-group difference in change from baseline to 12 months was not statistically significant (*p* = 0.053; see Supplementary Table S1). Adjusted sensitivity analyses did not demonstrate a significant overall or time-specific baseline-adjusted between-group difference in eGFR during follow-up (Supplementary Table S6).

For uACR, analyses were performed after log10 transformation because of the skewed distribution of values. No significant between-group differences in change from baseline in log10-transformed uACR were observed at either six or 12 months (*p* = 0.789 and *p* = 0.747, respectively; Figure 1B, Supplementary Table S2). Descriptive uACR values on the original scale showed wide variability and are provided in Supplementary Table S2. Adjusted sensitivity analyses likewise showed no significant overall or time-specific baseline-adjusted between-group difference in log10-transformed uACR during follow-up. A sensitivity analysis including the matched pairs affected by chronic antibody-mediated rejection yielded materially unchanged results (Supplementary Table S6).

Overall, the descriptive early decline in eGFR in the IG was not accompanied by a significant between-group difference in eGFR at 12 months; uACR likewise did not differ significantly between groups at 12 months.

#### 3.2.2 Metabolic parameters

**Figure 2:**
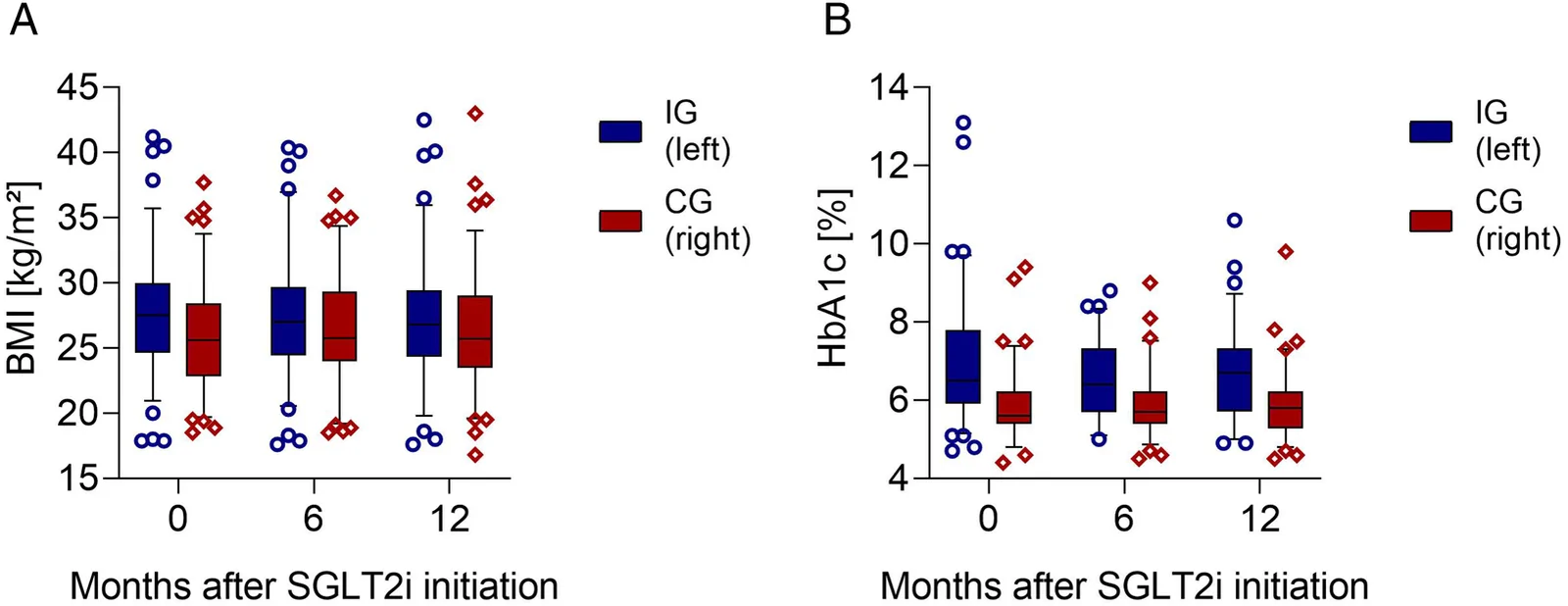
BMI and HbA1c over time. BMI **(A)** and HbA1c **(B)** over time in the IG (blue, left) and CG (red, right). Boxes indicate the interquartile range (25th–75th percentile), horizontal lines indicate the median, whiskers indicate the 5th–95th percentile, and individual points outside the whiskers represent outliers. BMI decreased in the IG and increased slightly in the CG; the overall adjusted between-group difference across follow-up remained statistically significant. HbA1c showed a favorable descriptive trajectory in the IG, but the between-group difference was not confirmed after multivariable adjustment.

Figure 2 illustrates metabolic parameters over time (see also Supplementary Tables S3 and S4). After six months, BMI decreased from 27.6 ± 4.5 to 27.4 ± 4.5 kg/m^2^ in the IG, and increased from 26.1 ± 4.2 to 26.4 ± 4.2 kg/m^2^ in the CG; the unadjusted between-group difference in change from baseline was significant (*p* = 0.020). After 12 months, the cumulative change from baseline was −0.4 kg/m^2^ (to 27.2 ± 4.6 kg/m^2^) in the IG and +0.2 kg/m^2^ (to 26.3 ± 4.5 kg/m^2^) in the CG; the unadjusted between-group difference in change from baseline remained significant (*p* = 0.001). In the IG, HbA1c decreased from 6.9 ± 1.5% at baseline (*n* = 88) to 6.5 ± 1% at six months (*n* = 71) and 6.7 ± 1.1% at 12 months (*n* = 68). In the CG, HbA1c remained largely unchanged (5.8 ± 0.8% with *n* = 85 at baseline, 5.9 ± 0.8% with *n* = 75 at six months, and 5.9 ± 0.9% with *n* = 80 at 12 months). In the unadjusted matched-cohort analysis, the between-group differences in HbA1c change were significant at both six months (*p* = 0.027) and 12 months (*p* = 0.013). Blood pressure did not differ significantly between groups during follow-up (*p* = 0.50 at six months and *p* = 0.49 at 12 months, see Supplementary Table S5 and Figure S1).

In the descriptive matched-cohort analysis, favorable trajectories were observed for both BMI and HbA1c, whereas blood pressure remained stable in both groups. In adjusted sensitivity analyses, the overall between-group difference in BMI remained statistically significant, whereas the descriptive between-group HbA1c difference was not confirmed after multivariable adjustment. Adjusted analyses of blood pressure did not materially alter the interpretation of the descriptive findings (Supplementary Table S6).

### 3.3 Potassium homeostasis and RAAS inhibitor use

Serum potassium remained stable over follow-up (Supplementary Tables S8 and S9, Figure S2). Hyperkalemia was associated with lower eGFR (*p* = 0.001). There was no evidence of a differential hyperkalemia trajectory according to SGLT2i exposure (group-by-time interaction *p* = 0.896). In patient-level analysis, higher eGFR was associated with lower odds of hyperkalemia (OR = 0.936 per mL/min/1.73 m^2^, *p* < 0.001). RAAS inhibitor persistence did not differ between groups (*p* = 0.301), whereas new RAAS inhibitor initiation was more frequent in the SGLT2i group [*p* = 0.001; see Supplementary Table S10 and Figure S3(B)].

### 3.4 Safety

The safety outcomes are summarized in Table 2. No statistically detectable between-group differences were observed for UTI, hospital admissions, cardiovascular events, or marked decline in graft function. One case of genital mycosis occurred in the IG. Two cases of graft loss occurred in the IG; according to the chart review, these were associated with pneumonia with septic shock and antibody-mediated rejection, respectively. In total, 12 patients discontinued SGLT2i therapy during follow-up, mostly due to mild UTI. One patient switched from dapagliflozin to empagliflozin and discontinued empagliflozin at the 12-month visit. No deaths occurred during the observation period.

**Table 2:** Safety outcomes during follow-up.

Clinical safety outcomes	IG events, n	IG participants affected, n/N (%)	CG events, n	CG participants affected, n/N (%)	p
Death	0	0/100 (0.0)	0	0/100 (0.0)	-
Graft loss	2	2/100 (2.0)	0	0/100 (0.0)	0.497
Cardiovascular events	4	4/100 (4.0)	5	5/100 (5.0)	1.000
Hospital admissions	59	37/100 (37.0)	90	44/100 (44.0)	0.388
eGFR decline > 30%	5	5/85 (5.9)	8	8/97 (8.2)	0.578
Urinary tract infections	29	18/96 (18.8)	47	25/92 (27.2)	0.224
Genital mycosis	1	1/100 (1.0)	0	0/100 (0.0)	1.000

Follow-up and treatment status	IG events, n	IG participants affected, n/N (%)	CG events, n	CG participants affected, n/N (%)	p
Discontinuation of SGLT2i	12*	12/100 (12.0)	N/A	N/A	-
Loss to follow-up	2	2/100 (2.0)	0	0/100 (0.0)	-

* One patient switched from dapagliflozin to empagliflozin and discontinued it at the 12-month visit.

Clinical safety outcomes in SGLT2i-treated (Intervention Group, IG) and control kidney transplant recipients (Control Group, CG) during follow-up. Total event counts and the number of participants experiencing at least one event are reported separately. Participants affected are presented as n/N (%), where N represents the number of participants with available follow-up information for the respective endpoint. Recurrent events within the same participant are included in the total number of events. *P*-values (Chi-square test or Fisher's exact test) refer to between-group comparisons of the proportion of participants experiencing at least one event, unless otherwise indicated. eGFR, estimated glomerular filtration rate, N/A = not applicable; SGLT2i = sodium-glucose cotransporter-2 inhibitors; UTI, urinary tract infection.

### 3.5 Effect of SGLT2 inhibitor-induced glucosuria on urinary albumin-to-creatinine ratio

**Figure 3:**
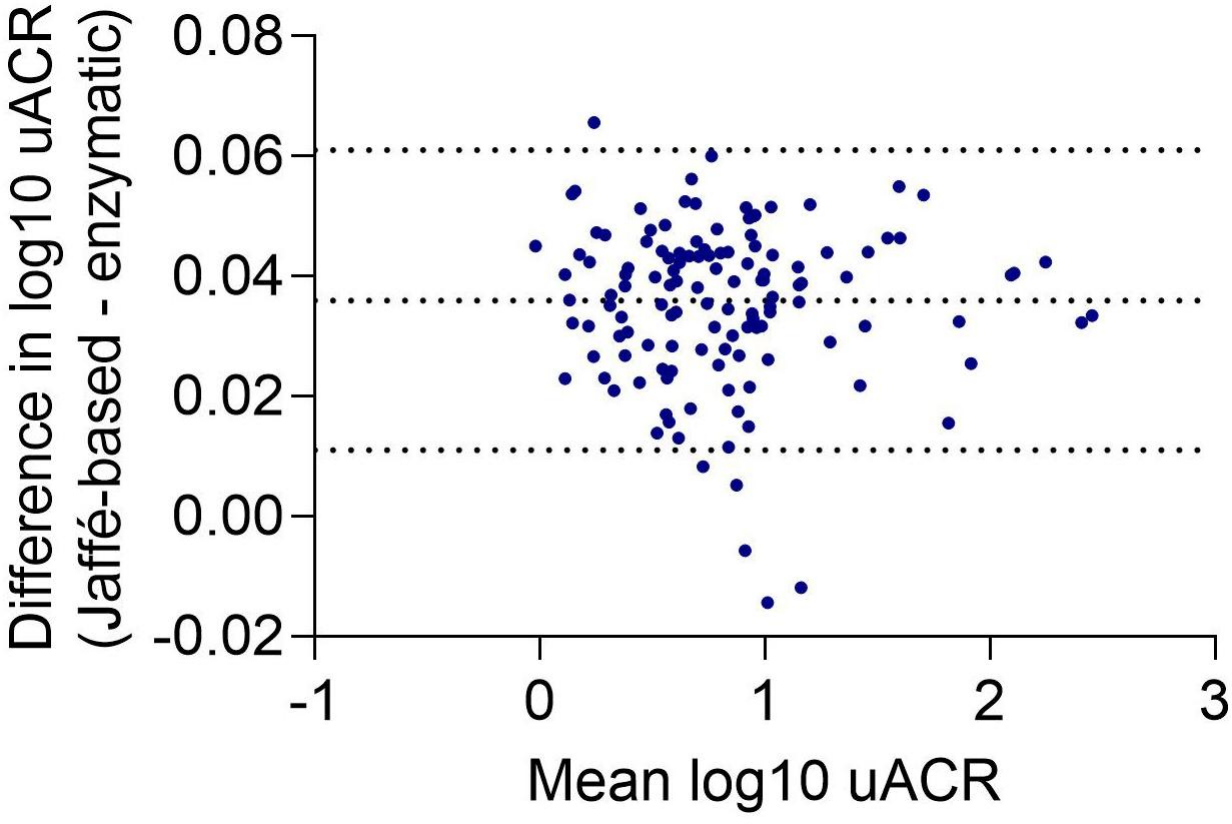
Bland-Altman analysis of uACR calculated using Jaffé-based and enzymatic urinary creatinine measurements. uACR values were log10-transformed because of skewed distribution. The *x*-axis represents the mean log10 uACR obtained by both methods, and the *y*-axis represents the difference between Jaffé-based and enzymatic log10 uACR. The central line indicates the mean bias, and the outer lines indicate the 95% limits of agreement. Positive values indicate higher calculated uACR when Jaffé-based urinary creatinine measurements are used.

Urinary creatinine concentrations measured by the Jaffé method were significantly lower than those obtained by the enzymatic method (see Figure 3). This resulted in an approximate 8.5% increase in calculated uACR when Jaffé-based creatinine values were used (*p* < 0.001). This difference led to assignment to a higher albuminuria stage in four of 128 samples: three shifted from A1 to A2, and one shifted from A2 to A3. Thus, while the analytical effect was statistically significant, its impact on clinical staging was limited.

## 4 Discussion

Given the limited data on SGLT2i in KTR, particularly in non-diabetic patients, we assessed the impact of SGLT2i therapy on metabolic parameters, graft-related endpoints, potassium-related safety, RAAS inhibitor use, and uACR measurement over 1 year in a matched cohort.

Descriptive matched-cohort analyses showed favorable trajectories of both BMI and HbA1c in SGLT2i-treated KTR. After multivariable adjustment, the between-group difference in BMI remained significant, whereas the descriptive between-group HbA1c difference was not confirmed. The BMI finding is broadly consistent with transplant-specific studies, including the randomized trial by Halden et al. and the multicenter observational cohort by Sánchez Fructuoso et al., both of which reported improved glycemic control and modest weight reduction after SGLT2i initiation in KTR (10, 11). Evidence syntheses similarly suggest that metabolic endpoints are among the most reproducible treatment-associated findings following kidney transplantation (9, 11, 12).

Our dataset does not allow conclusions regarding the mechanism underlying weight reduction. In non-transplant populations, both osmotic/volume-related and true metabolic effects have been described. However, this distinction could not be assessed here and should not be overinterpreted (22, 23).

An important consideration in interpreting the metabolic effects is the baseline imbalance between the intervention and control groups. Patients in the IG had a higher prevalence of DM, as well as higher baseline HbA1c and BMI, and more frequent RAAS inhibitor use, reflecting real-world prescribing patterns in which SGLT2i were preferentially prescribed to patients with a less favorable metabolic and renal risk profile. As these characteristics were not included in the matching algorithm, residual confounding by indication and regression to the mean cannot be excluded and may have influenced the magnitude of the observed treatment-associated differences. Consequently, the metabolic findings should not be interpreted as definitive evidence of a causal treatment effect. At the same time, these baseline differences reflect contemporary clinical practice and therefore enhance the real-world relevance of the cohort, as it represents the population in whom SGLT2i are currently most likely to be considered after kidney transplantation. Notably, despite the less favorable baseline metabolic profile of the IG, no excess in adverse renal, potassium-related, or infectious outcomes was observed during follow-up. Thus, while baseline imbalance limits causal inference regarding efficacy, the cohort remains informative for characterizing treatment-associated metabolic changes and short-term safety under routine clinical conditions. The main message of our study is therefore not a demonstrated short-term renoprotective effect, but rather a clinically relevant real-world profile: SGLT2i were associated with favorable metabolic changes and acceptable short-term safety, while potassium trajectories were stable and uACR interpretation was shown to be assay-sensitive under SGLT2i-associated glucosuria.

While the broader CKD literature provides a strong biological rationale for the use of SGLT2i, KTR were excluded from pivotal renoprotective trials. Consequently, the evidence base for KTR is distinct and currently supports the selective clinical use of SGLT2i rather than the direct extrapolation of the full cardiorenal benefit to this population (2–4, 12). Although SGLT2 inhibitors are known to mildly reduce blood pressure in CKD populations, we did not observe relevant between-group differences in blood pressure over time in this matched cohort. This finding is consistent with those of a recently published, prospective, placebo-controlled study (13). Therefore, changes in blood pressure are unlikely to explain the metabolic findings. However, changes in antihypertensive therapy remain a possible source of residual confounding in this retrospective design.

Regarding graft function, the descriptive early decline in eGFR observed in the IG was compatible with the known hemodynamic pattern after SGLT2i initiation. No significant between-group difference in eGFR was observed at 12 months, and our data do not establish whether longer-term renal benefit occurs in KTR (24, 25). Therefore, the key point is not that renal protection was demonstrated in our cohort, but rather that an initial fall in eGFR should not automatically be interpreted as harmful (9, 26). The underlying mechanism is generally attributed to increased distal sodium delivery, the restoration of tubuloglomerular feedback, and reduced intraglomerular pressure (24, 26, 27). Although this biological framework is relevant, our data do not allow us to draw mechanistic conclusions, and potential interactions with tacrolimus-based immunosuppression remain speculative in this cohort (28, 29). Since most patients received tacrolimus, subgroup analyses according to calcineurin inhibitor regimen were not feasible. Beyond hemodynamic mechanisms, anti-inflammatory, anti-fibrotic, oxidative stress-modulating, and potentially immunomodulatory effects of SGLT2i have been proposed as mechanisms that may be relevant to allograft function and rejection biology (30). However, this remains a hypothesis rather than an established clinical effect and our retrospective cohort was not designed to assess rejection activity, inflammatory pathways, or histological changes during follow-up.

Albuminuria showed wide variability and did not differ significantly between groups after log10 transformation in the principal analysis excluding recipients with chronic antibody-mediated rejection who typically exhibit very high albuminuria (5, 31). Results were materially unchanged in a sensitivity analysis including recipients with chronic antibody-mediated rejection. There are several possible explanations: limited statistical power, a short follow-up period, and biological heterogeneity of albuminuria in KTR. In this population, proteinuria may reflect chronic allograft injury, diabetes, recurrent or *de novo* glomerular disease, or ongoing alloimmune injury. Accordingly, our data neither exclude nor provide robust support for an antiproteinuric effect of SGLT2i. This cautious interpretation aligns with recent transplant-specific meta-analytic data that did not confirm a significant renal benefit overall, despite promising results in certain subgroups (9, 12, 32).

There are several factors that may explain why no significant differences in uACR or eGFR were observed between groups after 12 months. First, the limited follow-up of 12 months and the modest sample size may have reduced the ability to detect long-term renoprotective effects comparable to those observed in large prospective CKD trials. The present findings may therefore primarily reflect early metabolic and hemodynamic changes, whereas potential longer-term effects on albuminuria and graft function would require larger prospective transplant cohorts with extended follow-up (2, 3). Second, despite being matched for age, sex, and transplant duration, the IG had a less favorable metabolic risk profile at baseline. Third, KTR are exposed to transplant-specific determinants of graft function, including donor quality, rejection history, chronic allograft injury, and variable immunosuppression. These factors may dominate short-term eGFR and albuminuria trajectories.

The potassium analyses add a transplant-specific safety perspective to the study. In a recent routine-care target trial emulation, Bosi et al. reported lower hyperkalemia rates after SGLT2i initiation compared with DPP-4 inhibitors in patients with DM, without a corresponding improvement in RAAS inhibitor persistence. Our findings extend this question to KTR, but did not reproduce a clear potassium-lowering signal (20). This may reflect the specific pathophysiology of potassium handling after kidney transplantation, where graft function, calcineurin inhibitor exposure, RAAS blockade, DM, acidosis, and intercurrent illness may outweigh any modest kaliuretic effect of SGLT2i. The higher rate of new RAAS inhibitor initiation in the IG should be interpreted as confounded by indication rather than as a treatment effect. RAAS inhibitor persistence, however, did not differ significantly between groups, which argues against a clear treatment-related facilitation of ongoing RAAS blockade. The observed association between lower eGFR and hyperkalemia supports the physiological validity of the potassium analyses and suggests that the cohort captured an expected clinical pattern of impaired potassium handling in reduced kidney function. Taken together, these findings are exploratory and do not support a meaningful potassium-lowering effect of SGLT2i in KTR; importantly, however, they also do not suggest an increased potassium-related safety risk under real-world transplant conditions. A further limitation is that potassium values were available only at baseline, 6 months, and 12 months, so transient hyperkalemia episodes between visits may have been missed and the true hyperkalemia burden may be underestimated. Intermittent use of potassium-lowering therapies prescribed by primary care physicians or nephrologists, as well as unscheduled emergency department visits outside the tertiary care center, were not captured and therefore could not be investigated.

Importantly, we did not identify any clear short-term safety signals associated with SGLT2i use in KTR. There was no statistically detectable increase in marked eGFR decline, UTI, cardiovascular events, or hospitalizations in the IG, and genital mycosis was rare. Two graft losses occurred in the IG and were associated with pneumonia with septic shock and antibody-mediated rejection, respectively. Twelve patients discontinued SGLT2i therapy during follow-up, in several cases after minor increases in creatinine or infectious events, reflecting cautious real-world use in a population for which long-term experience remains limited. These observations are consistent with the available transplant-specific literature, including the randomized study by Halden et al., the multicenter cohort study by Sánchez-Fructuoso et al., and recent meta-analyses. Taken together, these studies suggest an acceptable short-term safety profile in selected recipients (9–13, 33, 34). However, rare adverse events, such as ketoacidosis, are difficult to assess in studies of this size. Patients with recurrent UTI (7 in IG vs. 11 in CG with ≥ 2 episodes) still warrant particular caution.

The uACR assay analysis is a clinically practical aspect of our study. We confirmed that SGLT2i-associated glucosuria can lower Jaffé-based urinary creatinine values and thereby increase calculated uACR compared with enzymatic measurement (14). However, reclassification to a higher albuminuria stage occurred in only 4 of 128 samples, indicating that the effect is analytically relevant but only rarely clinically decisive (35). This is relevant in transplant follow-up, where small changes in albuminuria may influence diagnostic reasoning, RAAS blockade decisions, and the threshold for further graft assessment. Enzymatic urinary creatinine assays should therefore be preferred where available, and if Jaffé-based methods are used, the assay type should be reported alongside uACR results.

The main strengths of this study are the matched-control design, which improves comparability relative to many prior uncontrolled transplant series, and the integration of metabolic, graft-related, potassium-related, safety, and laboratory-methodological endpoints within a single real-world transplant cohort. Several limitations must be acknowledged. The study is retrospective, single-center, and limited by modest sample size and short follow-up. A key limitation is residual confounding by indication. SGLT2i were preferentially prescribed to patients with higher metabolic risk, as reflected by higher baseline BMI, HbA1c, diabetes prevalence, and RAAS inhibitor use in the IG. Although matching was performed for age at transplantation, time since transplantation, and sex, it did not include DM, HbA1c, BMI, baseline proteinuria, serum creatinine, RAAS inhibitor use or pre-transplant listing time. Pre-transplant waiting time also differed between groups, indicating additional baseline heterogeneity that may reflect unmeasured differences between the cohorts. These limitations restrict causal inference and reduce generalizability, especially with regard to non-diabetic KTR.

In summary, SGLT2i therapy was associated with favorable metabolic changes, most consistently reflected by a reduction in BMI, and acceptable short-term tolerability. Renoprotective effects on albuminuria or graft function were not demonstrated over 1 year, and potassium analyses showed no evidence of treatment-related excess hyperkalemia. The descriptive early eGFR decline was compatible with the known hemodynamic dip associated with SGLT2 inhibition. Overall, these findings support the cautious use of SGLT2i in selected stable KTR, while highlighting the need for prospective studies addressing long-term graft outcomes, potassium-related safety, and assay-sensitive albuminuria assessment under SGLT2i-associated glucosuria.

## Data Availability

The raw data supporting the conclusions of this article will be made available by the authors, without undue reservation.
