# Supplementary material for: SGLT2 inhibitors in kidney transplant recipients: metabolic changes, potassium-related safety and implications for albuminuria assessment
Source: Front Transplant. 2026 Aug 18;5:1927374. doi: 10.3389/frtra.2026.1927374 (PMC13530008; doi:10.3389/frtra.2026.1927374)
Supplement: Supplementary file 1 [file Datasheet1.docx]

Supplementary Material

# 1 Supplementary Figures and Tables

| **eGFR (all patients)** | | | | |
| --- | --- | --- | --- | --- |
| Group | | Baseline | 6 months | 1 year |
| IG | Mean | 49.78 | 47.65 | 47.81 |
|  | N | 100 | 97 | 91 |
|  | Std. Deviation | 16.64 | 16.23 | 15.46 |
| CG | Mean | 47.43 | 47.27 | 46.23 |
|  | N | 100 | 99 | 99 |
|  | Std. Deviation | 17.86 | 18.54 | 18.29 |
| Total | Mean | 48.60 | 47.46 | 47.03 |
|  | N | 200 | 196 | 190 |
|  | Std. Deviation | 17.26 | 17.39 | 16.97 |
| **eGFR (with complete follow-up)** | | | | |
| Group | | Baseline | 6 months | 1 year |
| IG | Mean | 51.40 | 48.60 | 47.81 |
|  | N | 90 | 90 | 90 |
|  | Std. Deviation | 16.10 | 15.97 | 15.53 |
| CG | Mean | 47.48 | 47.48 | 46.17 |
|  | N | 98 | 98 | 98 |
|  | Std. Deviation | 17.82 | 18.52 | 18.38 |

**Supplementary Table S1.** Mean eGFR (in mL/min/1.73 m², CKD EPI) at baseline, 6 months and 1 year stratified by treatment group. All patients (n = 200) and with complete follow-up (n = 188).

| **Albuminuria (all patients without cAMR)** | | | | |
| --- | --- | --- | --- | --- |
| Group | | Baseline | 6 months | 1 year |
| IG | Mean | 149.01 | 152.03 | 115.52 |
|  | N | 85 | 81 | 78 |
|  | Std. Deviation | 424.51 | 427.25 | 228.98 |
| CG | Mean | 101.60 | 130.74 | 199.61 |
|  | N | 84 | 72 | 84 |
|  | Std. Deviation | 167.90 | 294.22 | 570.57 |
| Total | Mean | 125.44 | 142.01 | 159.12 |
|  | N | 169 | 153 | 162 |
|  | Std. Deviation | 323.41 | 369.62 | 441.23 |
| **Albuminuria (with complete follow-up and without cAMR)** | | | | |
| Group | | Baseline | 6 months | 1 year |
| IG | Mean | 133.56 | 132.09 | 116.53 |
|  | N | 77 | 77 | 77 |
|  | Std. Deviation | 412.74 | 380.48 | 230.30 |
| CG | Mean | 114.49 | 132.53 | 229.55 |
|  | N | 71 | 71 | 71 |
|  | Std. Deviation | 179.53 | 295.92 | 616.36 |
| **Albuminuria (log10-transformed, without cAMR)** | | | | |
| Group | | Baseline | 6 months | 1 year |
| IG | Mean | 1.74 | 1.72 | 1.74 |
|  | N | 85 | 81 | 78 |
|  | Std. Deviation | 0.51 | 0.53 | 0.46 |
| CG | Mean | 1.65 | 1.66 | 1.66 |
|  | N | 84 | 72 | 84 |
|  | Std. Deviation | 0.52 | 0.56 | 0.64 |
| Total | Mean | 1.69 | 1.69 | 1.70 |
|  | N | 169 | 153 | 162 |
|  | Std. Deviation | 0.52 | 0.54 | 0.56 |

**Supplementary Table S2.** Descriptive uACR values on the original scale and after log10 transformation (in mg/g creatinine) at baseline, 6 months and 1 year, stratified by treatment group. Inferential analyses were performed using log10-transformed uACR values. Recipients with chronic antibody-mediated rejection (cAMR) were excluded. The available-case baseline sample comprised 169 patients, and the complete-follow-up sample comprised 148 patients.

| **BMI (all patients)** | | | | |
| --- | --- | --- | --- | --- |
| Group | | Baseline | 6 months | 1 year |
| IG | Mean | 27.60 | 27.37 | 27.22 |
|  | N | 98 | 94 | 91 |
|  | Std. Deviation | 4.52 | 4.52 | 4.62 |
| CG | Mean | 26.10 | 26.42 | 26.31 |
|  | N | 98 | 96 | 99 |
|  | Std. Deviation | 4.18 | 4.22 | 4.53 |
| Total | Mean | 26.85 | 26.89 | 26.75 |
|  | N | 196 | 190 | 190 |
|  | Std. Deviation | 4.41 | 4.39 | 4.59 |
| **BMI (with complete follow-up)** | | | | |
| Group | | Baseline | 6 months | 1 year |
| IG | Mean | 27.48 | 27.35 | 27.08 |
|  | N | 86 | 86 | 86 |
|  | Std. Deviation | 4.37 | 4.55 | 4.52 |
| CG | Mean | 26.21 | 26.39 | 26.50 |
|  | N | 93 | 93 | 93 |
|  | Std. Deviation | 4.19 | 4.20 | 4.60 |

**Supplementary Table S3.** Mean BMI (in kg/m²) at baseline, 6 months and 1 year stratified by treatment group. All patients (n = 196) and with complete follow-up (n = 179).

| **HbA1c (all patients)** | | | | |
| --- | --- | --- | --- | --- |
| Group | | Baseline | 6 months | 1 year |
| IG | Mean | 6.89 | 6.53 | 6.65 |
|  | N | 88 | 71 | 68 |
|  | Std. Deviation | 1.51 | 0.99 | 1.14 |
| CG | Mean | 5.81 | 5.88 | 5.87 |
|  | N | 85 | 75 | 80 |
|  | Std. Deviation | 0.82 | 0.79 | 0.85 |
| Total | Mean | 6.36 | 6.20 | 6.23 |
|  | N | 173 | 146 | 148 |
|  | Std. Deviation | 1.33 | 0.95 | 1.06 |
| **HbA1c (with complete follow-up)** | | | | |
| Group | | Baseline | 6 months | 1 year |
| IG | Mean | 7.05 | 6.55 | 6.54 |
|  | N | 37 | 37 | 37 |
|  | Std. Deviation | 1.67 | 1.09 | 1.06 |
| CG | Mean | 5.84 | 5.90 | 5.87 |
|  | N | 47 | 47 | 47 |
|  | Std. Deviation | 0.87 | 0.76 | 0.70 |

**Supplementary Table S4.** Mean HbA1c (in %) at baseline, 6 months and 1 year stratified by treatment group. All patients (n = 173) and with complete follow-up (n = 84).

| **MAP (all patients)** | | | | |
| --- | --- | --- | --- | --- |
| Group | | Baseline | 6 months | 1 year |
| IG | Mean | 104.61 | 106.23 | 104.73 |
|  | N | 92 | 89 | 80 |
|  | Std. Deviation | 12.76 | 12.36 | 11.18 |
| CG | Mean | 102.01 | 102.22 | 101.55 |
|  | N | 93 | 88 | 91 |
|  | Std. Deviation | 12.12 | 11.38 | 10.46 |
| Total | Mean | 103.30 | 104.24 | 103.04 |
|  | N | 185 | 177 | 171 |
|  | Std. Deviation | 12.48 | 12.02 | 10.89 |
| **MAP (with complete follow-up)** | | | | |
| Group | | Baseline | 6 months | 1 year |
| IG | Mean | 104.50 | 107.90 | 104.83 |
|  | N | 69 | 69 | 69 |
|  | Std. Deviation | 12.95 | 11.53 | 10.53 |
| CG | Mean | 101.41 | 102.57 | 101.17 |
|  | N | 77 | 77 | 77 |
|  | Std. Deviation | 12.32 | 11.52 | 10.82 |

**Supplementary Table S5.** Mean MAP (in mmHg) at baseline, 6 months and 1 year stratified by treatment group. All patients (n = 185) and with complete follow-up (n = 146).

**Supplementary Figure S1.** MAP over time in the IG (blue, left) and CG (red, right). Boxes indicate the interquartile range (25th–75th percentile), horizontal lines indicate the median, whiskers indicate the 5th–95th percentile, and individual points outside the whiskers represent outliers. Individual data points are shown using different symbols for IG and CG.

| **Outcome, unit, n** | **Overall baseline-adjusted IG–CG difference during follow-up (95% CI), LMM** | **p** | **Group × time p** | **Baseline-adjusted IG–CG difference at 6 months (95% CI), ANCOVA** | **p** | **Baseline-adjusted IG–CG difference at 12 months (95% CI), ANCOVA** | **p** |
| --- | --- | --- | --- | --- | --- | --- | --- |
| eGFR, mL/min/1.73 m²,  *n: 165 \| 163 \| 159* | -1.685  (-4.062 to +0.692) | 0.163 | 0.333 | -2.295  (-4.768 to +0.178) | 0.069 | -1.372  (-4.181 to +1.436) | 0.336 |
| log10-uACR, principal analysis, log10 units,  *n: 161 \| 148 \| 157* | +0.035  (-0.078 to +0.149) | 0.538 | 0.982 | +0.031  (-0.088 to +0.151) | 0.605 | +0.041  (-0.098 to +0.179) | 0.565 |
| log10-uACR, sensitivity analysis (cAMR pairs included), log10 units,  *n: 185 \| 172 \| 180* | +0.034  (−0.076 to +0.144) | 0.545 | 0.538 | +0.043  (−0.073 to +0.160) | 0.463 | +0.031  (−0.104 to +0.165) | 0.653 |
| BMI, kg/m²,  *n: 189 \| 182 \| 182* | -0.473  (-0.875 to -0.071) | 0.021 | 0.101 | -0.299  (-0.738 to +0.141) | 0.181 | -0.666  (-1.178 to -0.155) | 0.011 |
| HbA1c, percentage points,  *n: 162 \| 120 \| 123* | +0.100  (-0.094 to +0.295) | 0.310 | 0.961 | +0.114  (-0.144 to +0.372) | 0.384 | +0.064  (-0.159 to +0.288) | 0.571 |
| MAP, mmHg,  *n: 176 \| 164 \| 156* | + 1.795  (-1.145 to +4.735) | 0.230 | 0.662 | +1.829  (-1.837 to +5.496) | 0.326 | +2.250  (-1.277 to +5.777) | 0.209 |

**Supplementary Table S6.** Adjusted sensitivity analyses of continuous clinical outcomes. Adjusted between-group differences are presented as intervention group (IG) minus control group (CG) with 95% confidence intervals (CI). Overall baseline-adjusted between-group differences during follow-up were estimated using linear mixed-effects models (LMM) including treatment group, follow-up time, the group-by-time interaction, the baseline value of the respective outcome, and clinically relevant baseline covariates. Time-specific baseline-adjusted between-group differences at 6 and 12 months were estimated using ANCOVA models with the same baseline covariates. Analyses were based on all available observations; no imputation of missing values was performed. The principal log10-uACR analysis excluded matched pairs affected by chronic antibody-mediated rejection; a sensitivity analysis including these pairs yielded materially unchanged results. Negative estimates indicate lower adjusted outcome values in the IG relative to the CG, whereas positive estimates indicate higher adjusted outcome values in the IG. n values are given for LMM | 6-month ANCOVA | 12-month ANCOVA and indicate the number of patients included in the respective model.

BMI = body mass index; CI = confidence interval; eGFR = estimated glomerular filtration rate; IG = intervention group; CG = control group; LMM = linear mixed-effects model; MAP = mean arterial pressure; RAAS = renin-angiotensin-aldosterone system; uACR = urine albumin-to-creatinine ratio.

| **Outcome** | **Covariates in addition to group/time** |
| --- | --- |
| BMI | baseline BMI, baseline eGFR, diabetes status, baseline RAAS inhibitor use |
| HbA1c | baseline HbA1c, baseline eGFR, diabetes status, baseline RAAS inhibitor use |
| eGFR | baseline eGFR, baseline BMI, baseline HbA1c, diabetes status, baseline RAAS inhibitor use |
| log10-uACR | baseline log10-uACR, baseline eGFR, diabetes status, baseline RAAS inhibitor use |
| MAP | baseline MAP, baseline eGFR, diabetes status, baseline RAAS inhibitor use |

**Supplementary Table S7.** Covariates included in the adjusted sensitivity analyses. For each continuous outcome, the table summarizes the baseline covariates included in the corresponding linear mixed-effects model and time-specific ANCOVA models. The LMMs included treatment group, follow-up time, the group-by-time interaction, the baseline value of the respective outcome, and the listed baseline covariates. The time-specific ANCOVA models at 6 and 12 months included treatment group and the same outcome-specific baseline covariates.

| **Serum potassium (all patients)** | | | | |
| --- | --- | --- | --- | --- |
| Group | | Baseline | 6 months | 1 year |
| IG | Mean | 4.32 | 4.24 | 4.27 |
|  | N | 100 | 97 | 91 |
|  | Std. Deviation | 0.59 | 0.43 | 0.51 |
| CG | Mean | 4.40 | 4.34 | 4.35 |
|  | N | 100 | 99 | 99 |
|  | Std. Deviation | 0.52 | 0.49 | 0.53 |
| Total | Mean | 4.36 | 4.30 | 4.31 |
|  | N | 200 | 196 | 190 |
|  | Std. Deviation | 0.56 | 0.47 | 0.52 |
| **Serum potassium (with complete follow-up)** | | | | |
| Group | | Baseline | 6 months | 1 year |
| IG | Mean | 4.29 | 4.24 | 4.27 |
|  | N | 90 | 90 | 90 |
|  | Std. Deviation | 0.54 | 0.43 | 0.51 |
| CG | Mean | 4.40 | 4.35 | 4.35 |
|  | N | 98 | 98 | 98 |
|  | Std. Deviation | 0.55 | 0.49 | 0.53 |

**Supplementary Table S8.** Mean serum potassium (in mmol/L) at baseline, 6 months and 1 year stratified by treatment group. All patients (n = 200) and with complete follow-up (n = 188).

**Supplementary Figure S2.** Comparison of serum potassium (A) and hyperkalemia prevalence (B, in %) in the intervention group (blue, left) and control group (red, right) over time. Boxes in (A) indicate the interquartile range (25th–75th percentile), horizontal lines indicate the median, whiskers indicate the 5th–95th percentile, and individual points outside the whiskers represent outliers. In (A), individual data points are shown using different symbols for IG and CG. Percentages in (B) are based on the available serum potassium measurements at each time point.

| **Hyperkalemia (> 5 mmol/L)** | | | | |
| --- | --- | --- | --- | --- |
| Group | | Baseline | 6 months | 1 year |
| IG | no | 89 | 92 | 83 |
|  | > 5 mmol/L | 11 | 5 | 8 |
| CG | no | 88 | 91 | 90 |
|  | > 5 mmol/L | 12 | 8 | 9 |
| Total | no | 177 | 183 | 173 |
|  | > 5 mmol/L | 23 | 13 | 17 |

**Supplementary Table S9.** Number of patients with hyperkalemia (> 5 mmol/L) at baseline, 6 months and 1 year stratified by treatment group. Based on available serum potassium measurements at each time point.

| **RAAS inhibitor use** | | | | |
| --- | --- | --- | --- | --- |
| Group | | Baseline | 6 months | 1 year |
| IG | None | 26 | 16 | 12 |
|  | RAASi | 72 | 78 | 77 |
|  | MRA | 2 | 3 | 5 |
| CG | None | 42 | 42 | 39 |
|  | RAASi | 52 | 53 | 56 |
|  | MRA | 6 | 4 | 4 |
| Total | None | 68 | 58 | 51 |
|  | RAASi | 124 | 131 | 133 |
|  | MRA | 8 | 7 | 9 |

**Supplementary Table S10.** Number of patients with RAAS inhibitor use (RAASi) and additional mineralocorticoid receptor antagonist (MRA) at baseline, 6 months and 1 year stratified by treatment group. Based on available medication data at each time point.

RAASi = ACE inhibitor or AT1 blocker; MRA = RAASi plus additional mineralocorticoid receptor antagonist.

**Supplementary Figure S3.** Percentage of patients with RAAS inhibitor persistence (A) and new RAAS inhibitor initiation (B) in the intervention group and control group. Panel A includes baseline RAAS inhibitor users with available follow-up medication data (IG, n = 71; CG, n = 57). Panel B includes only patients without RAAS inhibitor therapy at baseline (IG, n = 26; CG, n = 42).
